# 

**Published:** 1905-06-15

**Authors:** 


### ANNOUNCEMENTS.

THE NATIONAL SOCIETIES meet this year in Buffalo, N. Y. The Examiners meet July 24th. The National Dental Society July 25th, and the Faculties Association July 27th. The meetings will be held in the Iroquois Hotel, except the clinics, which will be held in the dental college.

THE INDIANA DENTAL SOCIETY will hold its meeting July 27th to 29th, in Indianapolis. This date conflicts with the National meetings and ought to be changed.

THE PENNSYLVANIA STATE MEETING occurs this year in Philadelphia on June 27th to 29th.

The Wisconsin State Meeting will be held this year in Oshkosh, July 18th to 20th.

---

The Michigan State Dental Association will meet this year in Detroit, July 10th, 11th and 12th. The meetings will be held in the dental department of the Detroit College of Medicine. This will undoubtedly be the largest meeting ever held by this association. An excellent program is being prepared and the Detroit dentists have planned to entertain the society in a manner that will be entirely satisfactory to every one.

The first session will be convened at 10 A. M, Monday, July 10th, when the President will deliver his address and the Directors and Trustees will make their reports. Such business matters as may require to be discussed or acted upon by the general body will be considered at this session.

The afternoon session will convene at 2 P. M., and will be given over to clinics on dental anesthetics. Every known method of dental anesthesia will be demonstrated by some one who has successfully used the method, and all appliances that are available will be shown and demonstrated. It will be a "painless symposium."

The evening session will be given up to the reading of papers on dental anesthesia and the discussion of the clinics.

*Tuesday Morning*: From eight to ten o'clock will be devoted to clinics on orthodontia, prophylaxis, oral and dental surgery. At 10 A. M. the members are invited to visit the drug manufacturing establishment of Parke, Davis & Co., where they will witness the processes of manufacture and partake of a slight luncheon. At half past twelve o'clock a large river steamer will call at Parke, Davis & Co's wharf and take the members with their wives and sweethearts for a ride to St. Clair Lake, and then down to Bois Blanc Island, where a banquet will be served at three o'clock by the Detroit dentists, after which the boat will return to Detroit. The evening session will be called at eight o'clock and will consist of papers on oral prophylaxis and pyorrhoea alveolaris.

*Wednesday Morning:* This session will be called at nine o'clock and will be given over to clinics on operative and prosthetic dentistry. All kinds of inlays, porcelain and gold, crowns, bridges, etc., and the various new and interesting methods in plate work. Exhibition of models of cavity preparation for fillings and inlay work, and an exhibition of methods used in testing amalgam alloys.

*Wednesday Afternoon:* Session will be given to the reading of papers on the subjects illustrated in the morning clinics and a discussion of the same.

It will be seen that the program is systematically constructed to bring before the profession all the newer and better methods of practical dentistry. Only nine papers will be read and these will be short and practical in purpose. A larger number of clinics will be given, and these by men who have become experts in their use. The place of meeting, time of the year and the pledged hospitality of the hosts will doubtless make this one of the greatest meetings ever held in the State. No Michigan dentist should miss it, and any non-resident of the State who wants the time of his life will not be unwelcomed. The usual fare and one-third round-trip rate will be given by all railroads and steamboat lines, and no better summer trip can be planned anywhere. There are many side trips that can be made from Detroit by those who may want to spend further time in sight-seeing of resting.

---

THE NEW JERSEY STATE DENTAL SOCIETY will meet as usual at Asbury Park, July 19th to 22nd. Many papers and more than fifty clinics, and a large exhibit is promised as an inducement to attend. Guests will be entertained and all will be privileged to bathe in and drink all the sea water they want.

THE WISCONSIN STATE BOARD OF EXAMINERS will meet in Milwaukee, June 26th, in the Wisconsin College of Physicians and Surgeons. For particular, address

DR. J. J. WRIGHT, *Sec.*
1818 Wells Bldg., Milwaukee.

THE SECTION ON STOMATOLOGY OF THE A. M. A. will meet in Portland, Ore., July 11th to 14th. Twenty-five papers are booked. This will be a grand chance to go to the Pacific Coast as the railway fares will be unusually low.

PERSONALS.—Dr. E. C. Kirk has been awarded a gold medal by the Odontological of Paris, France, for a research in dental science.—Dr. F. W. Shores, of Saginaw, Mich., has moved to Oakland, Cal.—Dr. F. C. Shober, of Cincinnati, was fined $3,500.00 by Judge Hosea, for mal-practice.—Dr. G. E. Hunt has become a litterateur. He is writing short stories for the popular magazines. We are not surprised as he has the talent. He will break into Congress one of these days.

THE AMERICAN SOCIETY OF ORTHODONTISTS, will hold its next meeting in Chicago, September 28th, 29th and 30th. This is a most unseasonable time for the meeting as it will preclude the presence of all college men, since the schools are all beginning their sessions at that time. A good program is in preparation and all who can attend will be amply repaid, as this is one of the best organizations we have, and its proceedings are always full of interest and value.

INTERSTATE DENTAL FRATERNITY.—The Board of Governors of the Interstate Dental Fraternity will convene for the annual business meeting of the order in Buffalo on Monday, July 24th. The annual banquet will occur during the week and due notice thereof will be sent to the members as soon as arrangements can be made and the exact date fixed. It is hoped that the fraternity will meet in large numbers on this occasion.

DR. R. M. SANGER,
*National Sec.*

THE OHIO STATE BOARD OF DENTAL EXAMINERS will meet to examine candidates for license to practice, at Columbus, O., June 27th to 29th, at the Hartman Hotel. Applications should be made before June 17th to Dr. H. C. Brown, Secretary, 185 E. State St., Columbus, O.

